# Healthcare-associated infections in home care settings: a systematic review of prevalence, risk factors, and prevention strategies

**DOI:** 10.3389/fpubh.2026.1871184

**Published:** 2026-06-24

**Authors:** Giustino Morlino, Francesca Volpi, Walter Priano, Giuseppa Granvillano, Giovanni Leonardo Briganti, Alessandro Belpiede, Eleonora Cimino, Gloria Spatari, Claudio Fiorilla, Antonio Antonelli, Stefania Borlini, Erika Alessandra Strangi, Michelangelo Mercogliano, Roberta Carestia, Maddalena Arcelli, Pier Paolo Russo, Fabio Ingravalle, Antonio Aprile, Dania Clemente, Francesco Leonforte, Giorgia Maria Ricciotti, Martina Chimienti, Andrea Calzavara, Enrica Frasson, Ruggero Geppini, Marta Pigozzo, Vittorio Grieco, Emilio Colarusso, Sara Fedrigucci, Simona Del Sorbo, Dario Genovese, Carla Fontana, Claudia Mosconi, Massimo Maurici

**Affiliations:** 1 Local Health Authority “Roma 1”, Roma, Italy; 2 Doctoral School in Nursing Sciences and Public Health, University of Rome “Tor Vergata”, Rome, Italy; 3 Department of Medicine and Surgery, University of Perugia, Perugia, Umbria, Italy; 4 Medical Management Unit, Azienda Ospedaliera Ospedali Riuniti Villa Sofia Cervello, Palermo, Italy; 5 Department of Medical and Surgical Sciences and Advanced Technologies "GF Ingrassia", University of Catania, Catania, Italy; 6 Department of Biomedical and Neuromotor Science, Alma Mater Studiorum, University of Bologna, Bologna, Italy; 7 Hospital Medical Management Unit, Scientific Institute for Research, Hospitalization and Healthcare–National Cancer Institute “Fondazione G. Pascale”, Naples, Italy; 8 Department of Life, Health and Environmental Sciences, University of L’Aquila, Italy; 9 Department of Health Sciences (DISSAL), University of Genoa, Genoa, Italy; 10 Department of Public Health, University of Naples “Federico II”, Italy; 11 School of Medicine, Università Vita-Salute San Raffaele, Milan, Italy; 12 Public Health Physician, University Hospital Polyclinic “G.Rodolico-San Marco”, Catania, Italy; 13 Department of Biomedical Sciences and Public Health, Section of Hygiene, Preventive Medicine and Public Health, Polytechnic University of the Marche Region, Ancona, Italy; 14 Local Health Authority “Roma 2”, Roma, Italy; 15 Hygiene and Public Health Unit, Department of Cardiac Thoracic and Vascular Sciences and Public Health, University of Padova, Padova, Italy; 16 Department of Hygiene and Public Health, University of Verona, Verona, Italy; 17 Department of Health Promotion, Mother and Child Care, Internal Medicine and Medical Specialties “G. D’Alessandro” (PROMISE), University of Palermo, Palermo, Italy; 18 Microbiology and Biobank Unit, National Institute for Infectious Diseases ‘L. Spallanzani’ IRCCS, Rome, Italy; 19 Department of Biomedicine and Prevention, University of Rome “Tor Vergata”, Rome, Italy

**Keywords:** antimicrobial resistance, HAIs, home care, prevalence, risk factors

## Abstract

**Background:**

Early hospital discharge has shifted complex care to domestic settings, increasing the risk of Healthcare-Associated Infections (HAIs). This review evaluates home care HAI prevalence, risk factors, and preventive challenges.

**Methods:**

Following PRISMA guidelines, a systematic search identified original studies on adult home care patients. Methodological quality was assessed using the RTI Item Bank and MMAT.

**Results:**

Ten studies were included in the narrative synthesis. Findings suggest that the overall prevalence of HAIs in the home care setting varies widely depending on reporting criteria and specific populations, also highlighting a critical epidemiological shift. In fact, up to 56% of home-managed HAIs were imported from previous hospitalizations thus actively introducing Multidrug-Resistant Organisms (MDROs)—such as ESBL-producing *E. coli* and *Clostridium difficile*—into the community setting. The prolonged use of invasive medical devices (e.g., urinary catheters, implantable ports), combined with severe chronic comorbidities like dementia, diabetes, and hypoalbuminemia, strongly amplifies infection risks. Furthermore, adherence to standard Infection Prevention and Control (IPC) protocols is frequently hindered by structural barriers, including a lack of cleanliness (85.4%) and space (77.1%). In this unregulated setting, the capability and education of informal caregivers act as primary determinants of infection outcomes.

**Conclusion:**

The home care environment is increasingly vulnerable to severe HAIs. Mitigating these risks requires the development of setting-specific IPC guidelines and the formal integration of caregiver education into public health policies.

**Systematic review registration:**

https://www.crd.york.ac.uk/PROSPERO/view/CRD42024594811, identifier PROSPERO (CRD42024594811).

## 1 Background

Healthcare-associated infections (HAIs) are a major global challenge for public health, with severe consequences for both patients and healthcare systems’ economic sustainability. According to the World Health Organization (WHO), HAIs are infections acquired during the care process, related to receiving health care, in any healthcare setting (e.g., acute care hospital, chronic care facility, ambulatory clinic, dialysis center, Ambulatory Surgery Center(ASC), home) that were neither present nor incubating at the time of admission (1, 2). Thus, home care and home hospice healthcare associated infections (HAIs) are those infections that were neither present nor incubating at the time of initiation of care in the patient’s place of residence (1, 2). As highlighted by the recent World Health Organization (WHO) Global Report on Infection Prevention and Control, Healthcare-Associated Infections (HAIs) pose a massive global burden: out of every 100 patients in acute-care hospitals, 7 in high-income countries and 15 in low- and middle-income countries acquire at least one HAI during their stay, with 1 in 10 affected patients dying from their infection (3). In Europe, the latest 2024 data from the European Center for Disease Prevention and Control (ECDC) estimates that over 4.3 million patients acquire an HAI each year in acute care hospitals alone (4). Globally, HAIs contribute to extended hospital stays and significant morbidity, generating a massive economic burden that consumes a significant proportion of hospital budgets and total healthcare expenses (5).

In recent years, the evolution of healthcare models has seen a significant shift from hospital-based care to community-based and home care settings, commonly referred to as “territorial care.” The increasing complexity of chronic patients and the aging population have driven this transition, prompting healthcare systems to adopt a model that reduces pressure on hospital facilities and promotes the integrated, continuous management of chronic diseases within the community (6). Furthermore, due to structural and financial pressures on acute care patients are being discharged from hospitals earlier (e.g., in a DRG-based prospective payment system), resulting in a potentially increase in the complexity and severity of home care patients. Over the last decade, the number of individuals receiving Home Health Care (HHC) has increased by more than 50% (7–9). While territorial care and home care offer benefits to patients such as a less stressful environment compared to hospital settings they also present new challenges. In a home care setting, the increased use of invasive medical devices and the management of complex therapies expose patients to a higher risk of HAIs (10).

Despite the growing importance of home care, scientific literature on HAIs in these settings remains surprisingly limited. While infections in hospitals are well documented and monitored, there is a lack of data regarding the incidence, risk factors, and prevention strategies for HAIs in home care (11). In Europe, only limited epidemiological data are available on HAIs in this specific setting, although leading organizations worldwide, such as the World Health Organization (WHO), acknowledge the increasing shift towards home-based care (12). There is a lack of data on the risks of infection for both patients and care workers in non-hospital settings (13, 14). Identifying patients who are at risk of infection is a key issue in community and home care settings (15). Limited research has largely focused on injection safety, while other precautions remain poorly studied (16). This information gap is concerning, given the increasing prevalence of home-based care and the need to prevent infections in a setting that is often less regulated than hospitals. As care delivery shifts from hospitals to patients’ homes, stringent prevention protocols and adequate training for healthcare personnel and caregivers become essential to reduce the risk of healthcare-associated infections (17). While previous systematic reviews have investigated infection prevalence in home health care [e.g., Shang et al., (18)] or focused exclusively on risk factors among older adults using mixed methods (19), our review provides a novel contribution by specifically addressing the adult population in the post-pandemic era. Furthermore, this study uniquely focuses on the importation of Multidrug-Resistant Organisms (MDROs), environmental barriers in home settings, and the critical role of informal caregivers in IPC strategies.

The aim of this systematic review is to gather scientific evidence by providing a comprehensive assessment of the incidence and impact of HAIs in home care settings. Specifically, this review seeks to highlight the clinical burden of HAIs in terms of health outcomes, identify specific risk factors, and examine the prevalence of involved pathogens (including Multidrug-Resistant Organisms - MDROs). Additionally, it aims to identify effective risk mitigation strategies and environmental challenges related to home care, in order to support a safe and sustainable community-based healthcare model. The findings of this review may serve as a starting point to develop evidence-based guidelines on infection prevention in home care settings.

## 2 Methods

### 2.1 Guidelines

This study aims to assess prevalence, risk factors and prevention strategies of healthcare-associated infections in the home care setting. PRISMA guidelines (20) were followed to conduct this systematic review. Inclusion and exclusion criteria were evaluated using the PICOS framework (21).

### 2.2 Eligibility criteria

The inclusion and exclusion criteria and PICOS framework are shown in Table 1. These criteria include studies published in English, which reported original research (e.g., cohort studies, cross-sectional studies, case–control studies, or qualitative surveys) or case reports, focusing on patients aged 18 years and older receiving care in a home care setting. The main objective was to evaluate the role and impact of various risk factors associated with the development of healthcare-associated infections in home care, based on current evidence. Systematic reviews and meta-analyses were excluded from the synthesis regardless of the language of publication. Furthermore, to capture the broadest range of observational and descriptive data, studies with or without a comparator group were considered equally eligible for inclusion.

**Table 1:** Inclusion and exclusion criteria.

PICOS framework	Inclusion criteria	Exclusion criteria
(P)opulation	Patients over 18 years old receiving formal assistance in a home care setting, reporting either infections strictly newly acquired during home care or healthcare-associated infections imported from a prior hospitalization but clinically managed within the home care setting;	Population in different settings (e.g., hospitals, long term care facilities); under 18 years old - pediatric population;
(I)ntervention/Exposure	Exposure to risk factors linked to the development of HAIs in home care assistance according to current evidence;	Setting without exposure to risk factors to develop HAIs; No risk factors mentioned;
(C)omparator	None;	None;
(O)utcome	To assess the role and weight of various risk factors on HAIs prevalence;	The study does not assess the relative importance of various risk factors (e.g., studies evaluating therapy or epidemiology);
(S)tudy design	Randomized Control Trials; Pre-post study; Quasi-experimental; Cohort study; Cross-sectional, prevalence of HAIs; Case–Control Study	Case report; Case series; Book chapter; Editorial; Commentary; Letter to editor; Systematic review; Meta-analyses;
Other criteria	Researches from 2013; Written in English; Peer-reviewed publication;	Full-text not available; Not written in English; Researches before 2013

### 2.3 Search strategy

The literature search was conducted encompassing articles published from 2013 up to January 2026. This comprehensive timeframe was selected to capture both historical trends and the most recent post-pandemic data. PubMed/MEDLINE was used as the primary database. Additional searches were performed in the secondary databases: Scopus and Web of Science (Clarivate). Duplicates were removed using Microsoft Excel. The search strategy utilized a combination of keywords and Boolean operators. The search strings used for this article are presented in Table 2.

**Table 2:** Strategy and terminology implemented for database search/Research string divided by domains.

Database	Search strategy/Search string
Medline/Pubmed	(home health* OR “home care” OR “home nursing” OR “domiciliary health care” OR (home AND care AND nurse*) OR (home AND care AND professional*) OR (home AND health AND agenc*) OR “Home Care Services”[Mesh]) AND (cross infection* OR superinfection* OR coinfection* OR HAI OR HAIs OR (healthcare AND associated AND infection*) OR (home AND care AND acquired AND infection*) OR (home AND care AND associated AND infection*) OR (home AND infection*) OR (secondary AND infection*) OR “Cross Infection”[Mesh] OR “Coinfection”[Mesh] OR “Superinfection”[Mesh]) AND (“hand hygiene” OR hygiene OR strateg* OR surveillance OR guideline* OR (alcohol AND based AND hand) OR “Hand Hygiene”[Mesh] OR “Hygiene”[Mesh] OR “Epidemiological Monitoring”[Mesh] OR “Guidelines as Topic”[Mesh])
Scopus	TITLE-ABS-KEY(“home health*” OR “home care” OR “home nursing” OR “home-care nurse*” OR “home care professional*” OR “home health agenc*” OR “domiciliary health care”) AND (“cross infection*” OR “healthcare associated infection*” OR “HAIs” OR “home care acquired infection*” OR “home care associated infection*” OR “home infection*” OR “superinfection*” OR “secondary infection*” OR “coinfection*”) AND (“alcohol based hand rub*” OR “alcohol based handrub*” OR “hand hygiene” OR “strateg*” OR “surveillance” OR “guideline*” OR “hygiene”)
Web of Science	((“home health*” OR “home care” OR “home nursing” OR “home-care nurse*” OR “domiciliary health care”) AND (“healthcare associated infection*” OR “HAIs” OR “cross infection*” OR “home care acquired infection*”) AND (“hand hygiene” OR “alcohol based hand*” OR “hygiene strategy*” OR “surveillance” OR “guideline*”))

### 2.4 Data items and outcomes definition

To ensure methodological clarity, we distinguished between two primary epidemiological categories of HAIs pertinent to our study: (1) strictly home-care-associated infections, defined as infections developed during home-based care without recent prior hospitalization; and (2) imported healthcare-associated infections, defined as infections acquired during a recent hospital stay but clinically managed within the home care setting. Community-associated infections lacking a clear link to professional medical care or homecare services were excluded. Lastly, to accurately reflect the infection burden that has to be managed by homecare providers, our synthesis includes both strictly home-care-associated and imported HAIs.

### 2.5 Study selection and data extraction

The study selection process was performed, after removing the duplicates, by a team of 12 reviewers divided into independent pairs, and consisted in two sequential phases: firstly, each pair of reviewers was assigned a specific subset of the retrieved records to independently screen titles and abstracts based on the predefined eligibility criteria; secondly, full texts of all potentially relevant articles were retrieved and independently assessed, by the corresponding pairs, for final inclusion. In both phases, final decision in case of disagreements, if not resolved through discussion and consensus within the pair of reviewers, was reached by consulting a third reviewer. Data extraction and study selection process was performed by using Microsoft Excel.

### 2.6 Quality assessment

Methodological quality and risk of bias of the included studies were independently evaluated by two reviewers. Disagreements were resolved through consensus or, if necessary, by consulting a third senior reviewer. To properly address methodological heterogeneity of the included studies, two distinct and validated appraisal tools were utilized:

The risk-of-bias assessment was conducted using two validated tools (RTI Item Bank and MMAT):

- For quantitative observational studies, RTI Item Bank scale (22) with its 13 core items was applied to evaluate selection, performance, attrition, detection biases, and confounding. To ensure objective and reproducible evaluation via RTI Item Bank, the predefined scoring approach applied to summarize the risk levels based strictly on the tool’s validated items. Specifically, based on the number of items exposing a study to a high risk of bias, studies with 2 or fewer risk-exposing items, were classified as Low Risk of Bias (Good Quality); those with 3 to 4 items as moderate Risk of Bias (Medium Quality); if 5 or more high risk items were retrieved, studies were considered at High Risk of Bias (Poor Quality).
- For studies that incorporated qualitative or mixed-methods designs, the Mixed Methods Appraisal Tool (MMAT, version 2018) was applied (23). MMAT Category 1 criteria were used to appraise purely qualitative studies, while MMAT Category 5 criteria were used for mixed-methods studies. Since MMAT methodological guidelines discourage the use of an overall numerical score, we opted instead to report item-level judgments (+ or -) to provide a more transparent and rigorous evaluation.

### 2.7 Protocol

The article protocol was registered on Prospero with ID: CRD42024594811.

## 3 Results

### 3.1 Study selection

**Figure 1:**
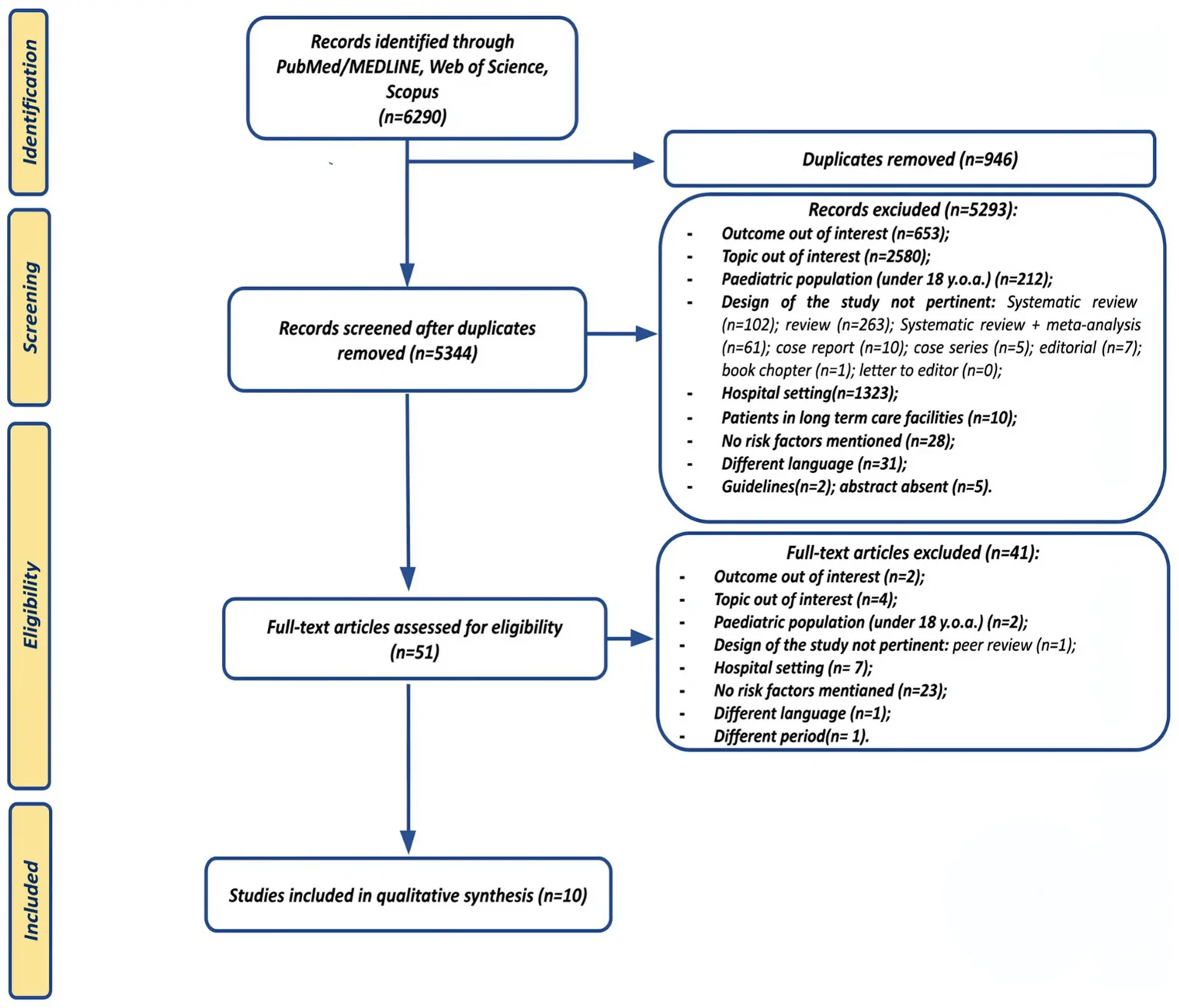
The flowchart of study selection (20).

The search and selection process is illustrated in the flowchart (Figure 1). A total of 6,290 studies were retrieved from the databases. After the title and abstract screening, 51 studies were selected for full-text evaluation, of which 41 were excluded.

### 3.2 Characteristics of the selected studies

The characteristics of the 10 included studies and summaries of their results are presented in Tables 3, 4. Although search was updated to early 2026, none of the studies published in 2025 or 2026 the inclusion criteria. Thus, studies were included from July 2015 up to March 2024.

**Table 3:** Study characteristics.

Author and Year (Country)	Study design and setting	Population
Brockhaus et al. (33) (Switzerland)	Cross-sectional survey/interviews. Home-healthcare organizations.	*n* = 162 home healthcare workers.
Dowding et al. (37) (USA)	Qualitative descriptive study (interviews). Home care agency.	*n* = 50 home care nurses.
Fawley et al., 2016 (30) (England)	Observational surveillance. Community vs. Healthcare settings.	*n* = 12,182 patients. (703 CA-CDI, 11,479 HA-CDI).
Lin et al. (31) (Taiwan)	Retrospective cross-sectional study. Home health care unit.	*n* = 598 patients. (Mean age: 81.9 years).
Maelegheer et al. (43) (Belgium)	Observational pilot study. Flemish home care nursing.	*n* = 711 patients. (52% > 75 years old).
Miliani et al. (26) (France)	National point prevalence survey. Home-based hospital care.	*n* = 5,954 patients. (Median age: 69 years).
Pichitchaipitak et al. (44) (Thailand)	Retrospective cohort. Home Parenteral Nutrition Center.	*n* = 72 adult patients. (Data spanning 2002–2014).
Raphael et al. (28) (USA)	Retrospective analysis. Urban safety-net healthcare system.	*n* = 6,287 bacteriuria episodes. (5,320\u00B0F, 967 M).
Shang et al. (36) (USA)	Retrospective analysis (OASIS data). Home care setting.	*n* = 128,163 patients. (Mean age: 77.1 years).
Song et al. (32) (USA)	Predictive modeling/NLP study. Large HHC agency.	*n* = 54,316 patients. (Median age: 67.6 years).

**Table 4:** Key findings.

Author and Year (Country)	Type of infection and pathogens	Key risk factors and comorbidities	Key findings and preventive measures
Brockhaus et al. (33) (Switzerland)	General IPC, Gastrointestinal, Respiratory. Focus on MDROs.	Environmental barriers: Lack of cleanliness (85.4%), lack of space (77.1%).	- Workers feel trained but struggle with MDRO information transfer, PPE use, and equipment disinfection. - Environmental limitations hinder IPC protocol adherence.
Dowding et al. (37) (USA)	General HAIs, UTIs, Wounds, Respiratory.	Caregiver availability, home cleanliness, patient age, diabetes, poor nutrition.	- Primary mitigation strategy is caregiver education (hand hygiene, cleaning bed-bound patients). - Use of risk-based protocols (e.g., MRSA kits) alongside universal precautions.
Fawley et al. (30) (England)	Gastrointestinal. (*Clostridium difficile*).	Age ≥ 65 years.	- CA-CDI cases were significantly more likely not to have received prior antibiotics vs. HA-CDIs (38.6% vs. 20.3%; *p* < 0.0001). - Ribotype 078 significantly higher in older adult CA-CDI cases.
Lin et al. (31) (Taiwan)	Respiratory. (Community-Acquired Pneumonia - CAP).	Nasogastric tube, Dementia, Anemia, Hypoalbuminemia.	Risk of CAP hospitalization significantly increased by: - Nasogastric tube use (OR 3.01) - Anemia (OR 2.37) - Chronic respiratory disease (OR 2.09) - Dementia (OR 1.94).
Maelegheer et al. (43) (Belgium)	Skin and soft tissue (9%), UTIs (4.5%), MDROs.	Medical devices (25% of patients, mostly urinary catheters). Diabetes (16%), Heart failure (15%).	- Skin infections surpassed UTIs. - 80% of diagnosed UTI patients required antibiotics. - Formal IPC training and MDRO screening programs are poorly implemented.
Miliani et al. (26)(France)	Overall HAIs (UTIs, Skin, BSI). Pathogens: MRSA (28.1%), ESBL.	41.9% of patients had at least one invasive device (urinary/vascular catheters).	- Overall HAI prevalence: 6.8%. - 56% of HAIs were imported from hospitals; only 35.5% were strictly home care-associated. -Antimicrobial prescription prevalence: 15.2%.
Pichitchaipitak et al. (44) (Thailand)	Catheter-Related Bloodstream Infections (CRBSIs). 26 pathogens (incl. *Candida* spp., *A. baumannii*).	Use of implanted port vs. tunneled catheter.	- CRBSI risk is significantly higher with implanted ports (IRR 4 to 1, *p*-value 0.007). - Disinfection with alcohol-based PVP-I led to significantly more infections compared to 2% CHG (IRR 4.1 to 1, *p*-value 0.001)
Raphael et al. (28) (USA)	UTIs. (ESBL-producing *E. coli*).	Age >65 years, Latinx ethnicity, Male gender.	- ESBL-*E. coli* is rapidly increasing in the community. - Age >65 and Latinx ethnicity are risk factors exclusive to the community/home setting, not the hospital.
Shang et al. (36) (USA)	Respiratory (1,880), UTIs (1,268), Wounds (1,039).	Urinary catheters, mobility issues, caregiver inability.	- Caregiver’s inability to safely perform medical procedures directly increases the risk of wound infections. - Advocates for predictive modeling using OASIS data.
Song et al. (32) (USA)	Wound infections.	Hypertension (59.8%), Diabetes (49.5%), Skin ulcers (45.2%), invasive devices.	- Highlights the critical burden of chronic comorbidities in wound infections. - Advocates the use of Machine Learning and NLP on clinical notes to preemptively identify high-risk patients.

### 3.3 Risk of bias in studies

The results of the evaluation performed to assess methodological quality and risk of bias are detailed in Tables 5, 6, respectively for quantitative observational studies and for qualitative and mixed-methods studies. By applying the methodology described in the previous paragraphs, the overall quality of the included literature has to be considered satisfactory. In fact, among the eight quantitative observational studies assessed via RTI Item Bank, six were classified as having a Low Risk of Bias (Good Quality), while the remaining two studies (Maelegheer et al. and Miliani et al.), which utilized a descriptive point-prevalence design, were classified as having a Moderate Risk of Bias (Medium Quality). For these two studies, moderate rating was primarily driven by features typical of cross-sectional designs, such as the inherent lack of control groups and multivariate confounding adjustments. Notably, none of the quantitative studies were classified as having a High Risk of Bias.

**Table 5:** RTI Item Bank assessment of study quality.

Author_Year	Q1	Q2	Q3	Q4	Q5	Q6	Q7	Q8	Q9	Q10	Q11	Q12	Q13	Risk of Bias	Overall Judgment
Fawley et al. (30)	+	+	+	+	+	+	+	−	NA	NA	+	+	+	1	Low Risk
Lin et al. (31)	+	+	+	+	−	+	+	−	NA	NA	+	+	+	2	Low Risk
Maelegheer et al. (43)	+	+	NA	+	−	+	+	−	NA	NA	NA	−	+	3	Moderate Risk
Miliani et al. (26)	+	+	NA	+	−	+	+	−	NA	NA	NA	−	+	3	Moderate Risk
Pichitchaipitak et al. (44)	+	+	+	+	−	+	+	−	+	+	+	+	+	2	Low Risk
Raphael et al. (28)	+	+	+	+	+	+	+	−	NA	−	+	+	+	2	Low Risk
Shang et al. (36)	+	+	+	+	+	+	+	−	NA	NA	+	+	+	1	Low Risk
Song et al. (32)	+	+	+	+	+	+	+	−	NA	NA	+	+	+	1	Low risk

(Legend: “+” = Criterion met / Low risk; “-” = Criterion not met/Exposes to risk of bias; “NA” = Not Applicable due to study design. Dowding et al. and Brockhaus et al. were evaluated using the MMAT tool and are excluded from this specific matrix).

**Table 6:** MMAT assessment of study quality.

Author_Year	Q1	Q2	Q3	Q4	Q5	Study design
Dowding et al. (37)	+	+	+	+	+	Qualitative (Category 1)
Brockhaus et al. (33)	+	+	+	+	+	Mixed Methods (Category 5)

(Legend: “+” = Criterion met. For Dowding et al., Q1-Q5 corresponds to MMAT items 1.1–1.5 for qualitative studies. For Brockhaus et al., Q1-Q5 corresponds to MMAT items 5.1–5.5 for mixed-methods studies).

Lastly, demonstrating rigorous data collection methods, robust thematic analyses, and highly effective integration of findings, both qualitative (Dowding et al.) and mixed-methods (Brockhaus et al.) studies met 100% of their respective MMAT category criteria.

### 3.4 Narrative synthesis

#### 3.4.1 Study characteristics and demographics

Characteristics of the 10 included studies, published between July 2015 and March 2024, are summarized in Tables 3, 4.

Regarding data collection periods, heterogeneity was observed: five of the included studies (50%) analyzed data collected from 2017 onward (Dowding et al., Lin et al., Maelegheer et al., Brockhaus et al., and Raphael et al., whose data collection extended until early 2020); the remaining five studies (50%) focused on earlier periods, between 2011 and 2014 (Fawley et al., Miliani et al., Shang et al., Song et al.). Notably, study performed by Pichitchaipitak et al. conducted the longest retrospective analysis, spanning from 2002 to 2014.

Demographic features exhibited significant variability among the body of evidence, although heavily focusing on older adults. Five studies (50%) focused heavily on older adults, with mean or median ages exceeding 65 years: Lin et al. (mean age 81.9 years), Shang et al. (mean age 77.1 years), Miliani et al. (median age 69 years), Song et al. (median age 67.6 years), and Fawley et al., who specifically highlighted incidence rates in patients aged ≥65 years. 52% of the cohort included in the study performed by Maelegheer et al. (total n° = 711) was over 75 years.

Finally, differences in infection rates between male and female population were reported in three of the included studies. Raphael et al. reported 5,320 *E. coli* bacteriuria cases among females compared to only 967 in males. A similar female predominance was observed by Fawley et al. for *C. difficile* infections and by Song et al. for wound infections.

#### 3.4.2 Prevalence and types of healthcare-associated infections

One of the key aspects emerging from narrative synthesis involved the true origin of infections managed in home care settings. Miliani et al. reported an overall prevalence of 6.8% of patients with at least one active HAI across a cohort of 5,954 individuals. Notably, 56% were infections “imported” from a previous healthcare facility, whereas only 35.5% were strictly associated with home-based care.

Regarding the spectrum of HAIs evaluated in home care settings, this included the following: urinary tract infections (UTIs), respiratory infections, skin/soft tissue and wound infections, gastrointestinal infections, and bloodstream infections (BSIs). Interestingly, UTIs represented a major burden in multiple cohorts, however, specific distributions varied. For instance, while Maelegheer et al. noted that skin and soft tissue infections (9%) prevailed on UTIs (4%) in their cohort, Shang et al. identified 1,268 UTI cases alongside 1,039 wound-site infections and 1,880 respiratory infections, retrieving a majority of respiratory infections rather than UTI cases. Also, respiratory infections, including community-acquired pneumonia (CAP), represented the primary focus for Lin et al. study, that performed a retrospective cross-sectional study at a home health care unit to evaluate the prevalence of community acquired pneumonia.

Bloodstream infections were specifically evaluated by Miliani et al., that identified 34 BSI cases and 12 cases of clinical sepsis, while Pichitchaipitak et al. focused exclusively on the prevalence of catheter-related bloodstream infections (CRBSIs) in port implanted versus tunneled catheter patients. Finally, gastrointestinal site infections were specifically investigated in studies performed by Fawley et al. and Brockhaus et al.

#### 3.4.3 Microbiological etiology and pathogen prevalence

Etiology widely varied across studies, and involved both Gram-positive / negative bacteria, and fungal pathogens also. Notably, Pichitchaipitak et al. isolated 26 different pathogens responsible of CRBSIs, including *Acinetobacter baumannii*, *Klebsiella pneumoniae*, *Staphylococcus aureus*, and fungal microorganisms such as *Candida* spp.

Raphael et al. analyzing 6,287 bacteriuria episodes, emphasized the growing burden of Extended-Spectrum Beta-Lactamase (ESBL)-producing strains, which was also taken into consideration, along with the role displayed by *Methicillin Resistant Staphylococcus Aureus* (MRSA), in the study performed by Miliani et al.

Finally, regarding gastrointestinal infections Fawley et al. provided an extensive analysis of *Clostridium difficile* infections (CDI) by comparing 703 Community-Associated (CA-CDI) cases with 11,479 presumed Healthcare-Associated (HA-CDI) cases, identifying distinct ribotype patterns: ribotypes 002, 020, and 056 predominated in CA-CDIs, whereas the epidemic ribotype 027 was significantly associated with HA-CDIs. CA-CDI cases were significantly more likely not to have received any antibiotic treatment in the 4 weeks prior to their episode compared to hospital-associated cases (38.6% vs. 20.3%; *p* < 0.0001). This finding indicates that in the community setting, *C. difficile* infections occurred frequently without prior antibiotic exposure.

#### 3.4.4 Medical devices, comorbidities, and quantifiable risks

The presence of invasive medical devices emerged as a critical, quantifiable risk factor. In fact, 41.9% of the patients included in the study performed by Miliani et al., which displayed an overall HAI prevalence of 6.8% and a total of 15.2% of antimicrobial prescription, had at least one invasive device such as urinary catheters and/or implantable venous access devices. Analyzing device specifics, Pichitchaipitak et al. demonstrated that the risk of CRBSIs is significantly higher in patients using an implanted port compared to a tunneled catheter, with an IRR (incidence rate ratio) of 4 events to 1, and a *p*-value of 0.007.

It is worth noting that, beyond the presence of medical invasive devices, overall health status and specific comorbidities can drastically amplify infection risks. In the study by Song et al., patients who developed wound infections exhibited exceptionally high rates of chronic comorbidities, including hypertension (59.8%), diabetes (49.5%), and pre-existing skin ulcers (45.2%), followed by peripheral vascular and pulmonary diseases. Similarly, Maelegheer et al. reported that 50% of home care patients included in their cohort presented significant comorbidities, mostly type I diabetes (16%) and venous insufficiency or heart failure (15%). The risk represented by comorbidities was further explored and quantified in the study performed through logistic regression by Lin et al. Interestingly, nasogastric tube use (OR 3.01, 95% CI 1.88–4.82), combined with hypoalbuminemia (OR 1.57, an indicator of malnutrition) and dementia (OR 1.94), significantly increased the risk of hospitalization for pneumonia. Finally, sociodemographic characteristics also played a role in infection risk in home care settings, as Raphael et al. revealed that for ESBL-producing *E. coli* bacteriuria, age over 65 and Latinx ethnicity represent risk factors exclusive to the community and home care setting.

#### 3.4.5 Infection prevention, specific protocols, and predictive models

Data relative to infection prevention evaluated the use of specific clinical protocols and the analysis of eventual environmental barriers. The qualitative study performed by Dowding et al. highlighted that home care nurses adapt care plans by implementing universal precautions for all patients and activating highly specific, risk-based protocols, such as the use of dedicated, exclusive kits for patients colonized with MRSA. Quantitative studies, such as the one performed by Pichitchaipitak et al., provided direct clinical evidence demonstrating that CRBSIs were significantly higher if disinfection of exit site was performed through alcohol-based povidone-iodine (PVP-I) rather than using 2% chlorhexidine gluconate (CHG), as shown in Table 4.

However, the implementation of standardized infection prevention and control (IPC) procedures clashes with structural limitations. Brockhaus et al. found that although healthcare workers felt prepared regarding hand hygiene and sharps management, major criticalities emerged in the application of protocols for equipment disinfection or in order to transfer information regarding patients colonized with multidrug-resistant organisms (MDROs). Furthermore, 85.4% of the workers cited a lack of cleanliness and 77.1% reported a lack of space as primary physical hurdles to protocol adherence. To preemptively address these complex variables, studies by Shang et al. and Song et al. advocated for the use of predictive risk models and Natural Language Processing (NLP) to proactively identify high-risk patients based on clinical notes, allowing for targeted preventive protocols to be activated before infections occur.

#### 3.4.6 The role of informal caregivers

Because healthcare providers have limited control over the home environment, the presence and capabilities of informal caregivers emerges as a key factor in ensuring safe care that can also play a role in infection control. In fact, Shang et al. explicitly ù highlighted that the informal caregiver’s inability to safely perform complex procedures delegated to the home setting, such as central line maintenance, wound care, or urinary catheter management, due to inadequate formal IPC training directly contributes to increase the risk for the patients to develop wound infections.

Also, Dowding et al. emphasized that nursing interventions heavily rely on caregiver education regarding hand hygiene, proper cleaning for bed-bound patients, and catheter maintenance as primary infection mitigation strategies.

## 4 Discussion

This systematic review highlights the critical, yet poorly explored, burden of Healthcare-Associated Infections (HAIs) in the home care setting that, unlike the well-documented nosocomial environment, presents its unique epidemiological challenges as the dynamics of non-hospital care are rapidly changing. As of today, with relative increasing frequency, patients with complex needs and invasive devices are discharged early (18), thus continuously crossing the boundary between hospital and home pathogens.

The domestic environment, historically considered at low-risk to develop HAIs, is no longer shielded from the migration of resistant strains into the community (24, 25), as heavily emphasized both by the European Center for Disease Prevention and Control (ECDC) and recent literature regarding the presence of Multidrug-Resistant Organisms (MDROs) in the home care setting. This aspect is strongly corroborated by our findings: Miliani et al. (26) demonstrated that 56% of HAIs managed at home were actually “imported” from prior hospital admissions, thus introducing hospital-adapted pathogens into the community by discharging the patient (27). Furthermore, Raphael et al. (28) documented a significant burden of ESBL-producing *E. coli* bacteriuria in community-dwelling older adults, thus underlining the need for specific behavioral interventions, such as multi-faceted interventions, face to face or online training and educational interventions, catheter removal protocols and cathether management training (29). Fawley et al. (30) revealed that community-associated *Clostridium difficile* infections (CA-CDI) frequently occur without the preceding antibiotic exposure typically required to trigger the disease in hospitals, suggesting alternative environmental reservoirs of pathogens in the community.

A 2025 meta-analysis performed by Liljas et al. (19) highlighted the critical nature of this setting by quantifying the role of specific clinical risk factors—such as indwelling catheters and neurological comorbidities— though exclusively taking the older adult home care population into consideration. While their findings firmly establish the clinical vulnerabilities of the geriatric population, our review expands this perspective by analyzing the adult home care population as a whole and highlighting the microbiological shift occurring in this setting. Beyond individual clinical risks, our synthesis reveals that the epidemiological mechanics of HAIs in outpatient settings heavily depend on the transfer of MDROs from acute care facilities to the home environment. In fact, the growing risk of HAIs in the community is intrinsically tied to the ongoing temporal trend of de-hospitalization, shifting complex medical care and invasive devices to the home environment. In this context, informal caregivers (i.e., family members without professional medical background) serve as the primary line of defense. However, they face a dual-edged reality: while essential for patient management, they are hindered by severe clinical (e.g., lack of specific IPC training) and environmental barriers (e.g., inadequate home layout, sharing of limited spaces, or lack of proper sanitation supplies), typical of a less-controlled setting. To properly discuss the critical impact of this setting on infection risk both in healthcare professionals and in informal caregivers, we must highlight that the prolonged use of invasive medical devices, into this environment, can potentially represent a primary driver of infection. Firstly, as demonstrated both by Lin et al. (31) and Song et al. (32), the presence of invasive devices (such as nasogastric tubes) and, contemporarily, of chronic comorbidities (e.g., dementia, diabetes, and hypoalbuminemia) is strongly associated with critical adverse outcomes: whereas in hospitals, device management is governed by strict, standardized protocols, in the home setting, as reported by Brockhaus et al. (33), these protocols are frequently compromised by severe environmental barriers, with 85.4% of healthcare workers citing a lack of cleanliness (34) and 77.1% reporting a lack of space as major obstacles.

Finally, due to the fact that homecare is a setting in which healthcare professionals cannot appropriately apply continuous monitoring of patients into their homes (35), the daily burden of infection prevention and control (IPC) largely falls on informal caregivers who cannot safely perform medical procedures, thus significantly increasing the risk of cross-contamination and wound infections (36). Conversely, when properly educated on IPC measures such as hand hygiene and device maintenance, caregivers serve as the primary line of defense against infections (37–39).

The aspect that clearly emerges from our synthesis is that to mitigate these growing risks, IPC strategies can no longer be applied solely into hospital-settings. There is an urgent need to develop and implement standardized, setting-specific IPC guidelines tailored to the structural and environmental realities of domestic care (40, 41). Also it is of utmost importance for future public health policies to prioritize the active integration and formal education of informal caregivers, transforming them from passive bystanders into active participants in infection prevention (42).

### 4.1 Limitations

This systematic review has some limitations that must be acknowledged. First, the search strategy utilized strictly ‘AND’ Boolean operators to combine home care, infection, and IPC strategies related terms. Inadvertently, this approach may have excluded prevalence or risk factor studies that did not explicitly use terminology related to guidelines, hand hygiene, or surveillance systems. Methodological heterogeneity among the included studies must also be taken into consideration, as it ranged from point-prevalence surveys to qualitative interviews and predictive modeling. This aspect, combined with the lack of a universally standardized definition for HAIs, specifically tailored to the home care setting across different countries, precluded the possibility to perform a quantitative synthesis. Furthermore, a significant geographic bias was detected in the available literature as the included studies were exclusively performed in high-income or upper-middle-income countries. Thus, findings regarding device availability, IPC protocols, and environmental barriers may not be generalizable to low- and middle-income countries, where home care dynamics and resources drastically differ. Finally, the reliance on retrospective data and clinical notes in several studies may have led to an underreporting of true infection rates, as mild HAIs treated directly at home without hospitalization often escape formal surveillance networks. Regarding the study limitations,

## 5 Conclusion

This review, by highlighting the transition of HAIs from acute hospitals to the home care setting, points towards a significant shift in public health paradigms. Findings suggest that the hidden burden of HAIs—often underreported or misclassified outside formal clinical environments such as hospitals—is exacerbated by the importation of MDROs and relies also on the role of informal caregivers.

To fully understand and effectively address these risks, there is an urgent need to develop and implement standardized, specific IPC guidelines tailored to the homecare setting. To foster these needs, future public health policies should invest into the active integration and formal education of caregivers, and, ultimately, strengthen surveillance systems specifically designed for the home care setting.

## Data Availability

The original contributions presented in the study are included in the article/Supplementary material, further inquiries can be directed to the corresponding author/s.
